# Multiparametric cardiovascular magnetic resonance imaging in acute myocarditis: a comparison of different measurement approaches

**DOI:** 10.1186/s12968-019-0568-x

**Published:** 2019-08-29

**Authors:** Darius Dabir, Thomas M. Vollbrecht, Julian A. Luetkens, Daniel L. R. Kuetting, Alexander Isaak, Andreas Feisst, Rolf Fimmers, Alois M. Sprinkart, Hans H. Schild, Daniel Thomas

**Affiliations:** 1 Department of Radiology, University of Bonn, Sigmund-Freud-Str. 25, 53127 Bonn, Germany; 2 Department of Medical Biometry, Computer Science, and Epidemiology (IMBIE), University of Bonn, Bonn, Germany

**Keywords:** Myocarditis, Mapping, ECV, Measurement approach, Accuracy

## Abstract

**Background:**

Myocardial T1 and T2 mapping are reliable diagnostic markers for the detection and follow up of acute myocarditis. The aim of this study was to compare the diagnostic performance of current mapping measurement approaches to differentiate between myocarditis patients and healthy individuals.

**Methods:**

Fifty patients with clinically defined acute myocarditis and 30 healthy controls underwent cardiovascular magnetic resonance (CMR). Myocardial T1 relaxation times, T2 relaxation times, left ventricular (LV) function, T2 ratio, early gadolinium enhancement ratio, and presence of late gadolinium enhancement (LGE) were analysed. Native T1 and T2 relaxation times, as well as extracellular volume fraction (ECV) were measured for the entire LV myocardium (global), within the midventricular short axis slice (mSAX), within the midventricular septal wall (ConSept), and within the remote myocardium (remote). Receiver operating characteristics analysis was performed to compare diagnostic performance.

**Results:**

All measurement approaches revealed significantly higher native T1 and T2 relaxation times as well as ECV values in patients compared to healthy controls (*p* < 0.05 for all parameters). The global measurement approach showed highest diagnostic performance regarding all mapping parameters (AUCs, native T1: 0.903, T2: 0.847, ECV: 0.731). Direct comparison of the different measurement approaches revealed significant differences in diagnostic performance between the global and the remote approach regarding T1 relaxation times and ECV (*p* = 0.001 and *p* = 0.002 respectively). Further, the global measurement approach revealed significantly higher T1 relaxation times compared to the ConSept approach (AUCs: 0.903 vs. 0.783; *p* = 0.003) and nearly significant differences compared to the mSAX approach (AUC: 0.850; *p* = 0.051).

T2 relaxation times showed no significant differences between all measurement approaches (*p* > 0.050 for all parameters).

**Conclusions:**

Native T1 and T2 mapping allow for accurate detection of acute myocarditis irrespective of the measurement approach used. Even measurements performed exclusively within remote myocardium allow for reliable detection of acute myocarditis, demonstrating diffuse involvement of disease despite a mostly regional or patchy distribution pattern of visible pathologies. The global measurement approach provides the overall best diagnostic performance in acute myocarditis for both T1 and T2 mapping.

**Electronic supplementary material:**

The online version of this article (10.1186/s12968-019-0568-x) contains supplementary material, which is available to authorized users.

## Background

Myocarditis is defined as inflammatory disease of the myocardium, diagnosed by established histological, immunological, and immunohistochemical criteria [1]. Acute myocarditis accounts for up to 81% of patients presenting with chest pain, elevated troponin, but unobstructed coronary arteries and accounts for 12% of sudden cardiac deaths in young adulthood [2, 3]. Due to a mostly non-specific clinical presentation, the clinical diagnosis of disease remains challenging. Cardiovascular magnetic resonance (CMR) imaging has emerged as the most accurate non-invasive imaging modality for evaluation of cardiomyopathies and myocarditis in particular. CMR not only represents the gold-standard for assessment of left ventricular (LV) and right ventricular (RV) function, but also allows for characterization of tissue abnormalities such as myocardial edema and/or fibrosis.

CMR based diagnosis of acute myocarditis is based on the “Lake Louise” criteria (LLC), which were recently supplemented by quantitative imaging parameters [4]. T1 and T2 mapping including extracellular volume (ECV) fraction are quantitative techniques which allow for accurate quantification of myocardial fibrosis and edema [5, 6]. Both, T1 and T2 mapping are proven reliable diagnostic tools enabling detection and follow up of acute myocarditis and offer the prospect of objective analysis while overcoming limitations of semiquantitative imaging techniques used for the LLC (e.g. artefacts and concomitant involvement of skeletal muscle) [7–12]. Several measurement approaches are currently used to evaluate T1 and T2 relaxation times. Of these, the midventricular short axis (mSAX) approach (including the analysis of the entire LV short axis slice) and the global approach (including the analysis of myocardium within the whole apical, midventricular, and basal short axis slice) represent the most commonly used methods. Recently, midventricular sept wall (ConSept) (measurement of T1 relaxation times within the midventricular septal wall) was proposed as the standardized approach to distinguish health from disease in diffuse myocardial involvement [13].

The aim of this study was to compare the diagnostic performance of these currently employed approaches to differentiate myocarditis patients from healthy individuals. Since the mSAX and global approach typically include obviously affected myocardial segments, the true value of quantitative native T1 and T2 relaxation time assessment might be overestimated. Thus, we additionally investigated the diagnostic value of myocardial mapping when only performed in remote myocardium.

## Methods

### Study population

This prospective study was approved by the local ethics committee and all subjects gave written informed consent before CMR imaging.

Between 03/2014 and 05/2018 50 patients fulfilling the diagnostic criteria for clinically suspected myocarditis by the European Society of Cardiology [14] were enrolled in this observational cohort study. Patients neither had history of cardiac disease, nor cardiac risk factors (e.g. diabetes mellitus or smoking). Thirty-three patients (66%) suffered from arrhythmias and/or electrocardiogram (ECG) abnormalities at the time of their hospitalization. The most common forms were ST-elevation (*n* = 14), supraventricular tachycardia (*n* = 9), and RV conduction block (*n* = 6). None of the patients suffered a major adverse cardiac event within the next 6 months of active surveillance. The control group consisted of 30 healthy subjects and outpatients referred for nonspecific thoracic pain who did not show structural abnormalities on CMR. Outpatients had no medical history and did not take regular medication. Clinical tests including Holter, cardiac enzymes, echocardiography, and cardiac stress test as well as clinical follow-up were unremarkable. Exclusion criteria for all subjects were contraindications to contrast-enhanced CMR imaging (implantable devices, cerebral aneurysm clips, cochlear implants, severe claustrophobia, chronic kidney disease).

### CMR protocol

All examinations were performed on a 1.5 T CMR scanner (Ingenia; Philips Healthcare, Best, the Netherlands) using a 32-channel torso coil with digital interface for signal reception. Sequences were acquired according to the updated Society for Cardiac Magnetic Resonance recommendations [15]:

*Functional imaging* consisted of ECG-gated balanced steady state free precession cine sequences acquired during breath hold in horizontal long axis (HLA), vertical long axis (VLA), LV outflow tract (LVOT), and short axis (SAX), the latter covering the whole LV from apex to base.

Detection of inflammation-induced *myocardial edema* was performed using black blood T2- short tau inversion recovery (STIR) imaging in SA, VLA, and transverse orientation.

*Inflammation-induced hyperaemia* was assessed using transverse free-breathing T1 weighted images prior and <3 min after intravenous injection of a double dose (0.2 mmol per kilogram body weight) of extracellular contrast agent (Gadovist, Bayer Healthcare, Berlin, Germany) as previously described [8].

*Myocardial fibrosis and scarring* using the late gadolinium enhancement (LGE) technique was performed 15 min after contrast agent injection using T1 weighted segmented inversion-recovery gradient-echo sequences acquired in HLA, SAX, and VLA. The correct inversion time was determined using the Look-Locker technique.

In addition to the standard CMR protocol native and post-contrast T1 and T2 mapping were performed. T2 maps were acquired prior to contrast agent injection in end-diastole in 3 short axis slices (apical, mid, basal) using a multiecho dataset based on two established CMR techniques (gradient spin-echo (GraSE)), as previously described [16]. Before and 10 min after administration of contrast agent T1 maps were obtained in end-diastole in 3 corresponding short axis slices (apical, mid, basal) using a balanced steady-state free precession based 3–3-5 modified Look-Locker inversion recovery scheme, as previously described [17].

### Image analysis

Images were evaluated by two radiologists with 9 and 6 years of CMR experience. Functional analysis (LV end diastolic volume (LVEDV), LV ejection fraction (LVEF), interventricular septal thickness (IVST)) was determined offline using dedicated software (ViewForum, Philips Healthcare). LVEDV and LVEF were quantified manually by tracing the endocardial borders. Papillary muscles were included in the LV volume. Presence of myocardial edema was evaluated both visually and by comparing the signal intensity of the LV myocardium in STIR-weighted SA images against the signal intensity of a reference region within the adjacent skeletal muscle in the same slice (T2 ratio), as previously described [18]. The early gadolinium enhancement ratio (EGEr) for detection of inflammation-induced hyperaemia was determined as previously described [19]. For T2 ratio and EGEr the mean value of 2 respectively 3 measurements were used for statistical analysis.

**Fig. 1:**
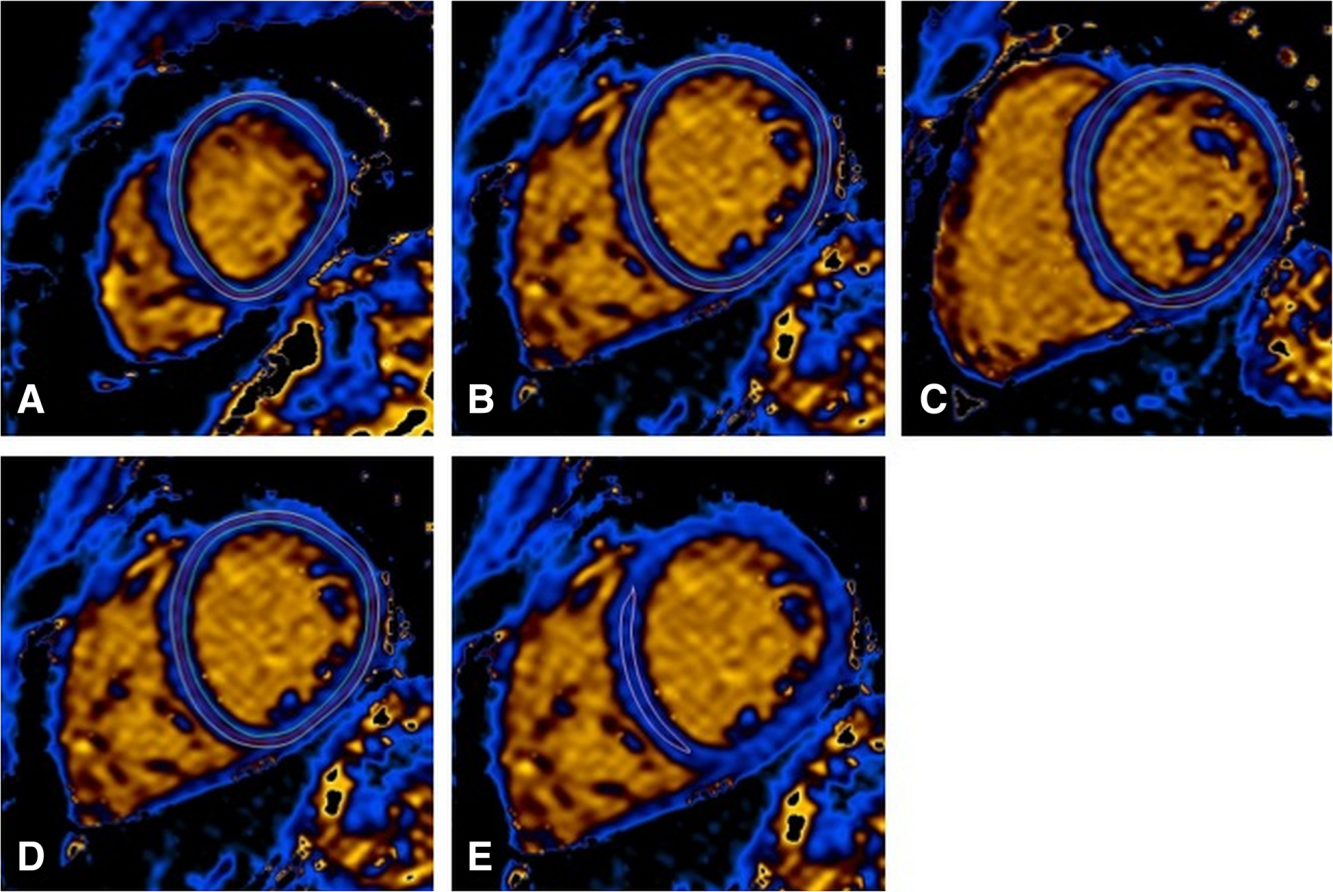
Example of the different measurement approaches. Colour encoded T1 maps in short axis (SAX) orientation with examples of different measurement approaches. (**a**-**c**) Global approach with the region-of-interest (ROI) including the whole apical (**a**), midventricular (**b**), and basal (**c**) SAX slice (in the latter case at the transition from midventricular to basal). (**d**) mid short axis (mSAX) approach with ROI placement within the midventricular short axis slice. (**e**) midventricular septal wall (ConSept) approach with ROI placement conservatively within the midventricular septum, taking care to avoid “contamination” with signal from the blood pool

**Fig. 2:**
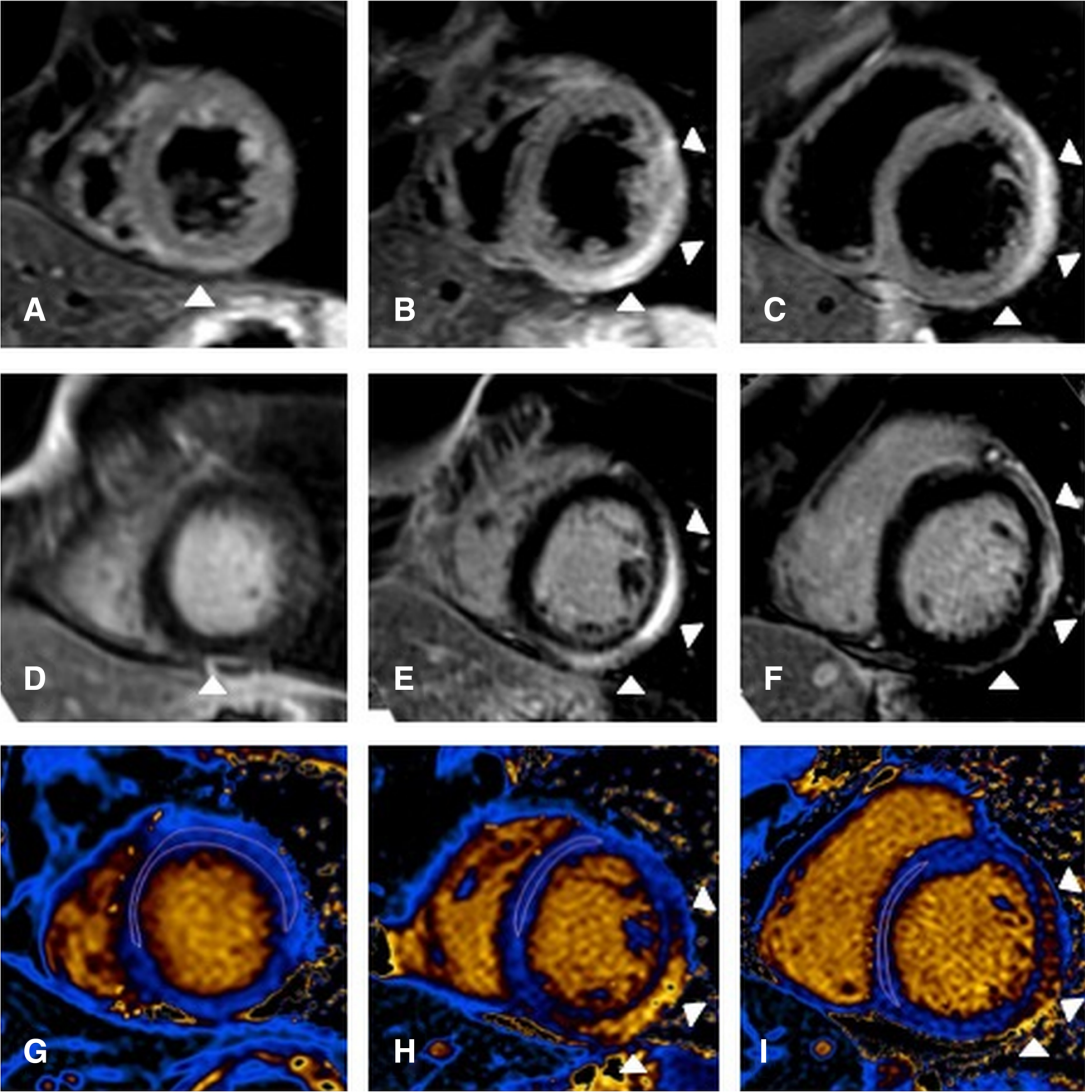
Remote measurement approach. 30 year-old patient with acute myocarditis, markedly located within the midventricular and basal lateral wall (arrow heads). The figure shows short tau inversion recovery (STIR) images (**a**-**c**) and late gadolinium enhancement (LGE) images (**d**-**f**), as well as native T1 maps (**g**-**i**) in apical (**a**, **d**, **g**), midventricular (**b**, **e**, **h**), and basal (**c**, **f**, **g**) SA orientation. The remote measurement approach is shown within the T1-maps (**g**-**i**) where a ROI was placed within visually unaffected segments and at a safe distance from affected ones

T1 and T2 maps were evaluated using dedicated software (Philips Intellispace 9, Philips Healthcare). Automatic motion correction was performed prior to analysis in all maps. For measurement of T1 and T2 relaxation times different approaches were used: including the complete apical, midventricular, and basal SAX slice (global), including the complete mSAX, within the midventricular septal wall (ConSept), and within the remote myocardium (remote = normokinetic segments with no visual edema or presence of LGE). For the assessment of global, mSAX, and remote T1 and T2 relaxation times, endo- and epicardial contours of the LV were traced while excluding epicardial fat, pericardium, and blood from analysis. ConSept measurements were performed by placing a region of interest (ROI) conservatively within the midventricular septal wall taking care to avoid contamination with the blood pool, as previously described [13] (Figs. 1 and 2).

### Statistical analysis

Statistical analysis was performed using SPSS software (version 24, Statistical Package for the Social Sciences (SSPS), International Business Machines, Inc., Armonk, New York, USA) and MedCalc 11.0 (MedCalc Software bvba, Ostend, Belgium). Patients’ characteristics are presented as mean ± standard deviation or as absolute frequency. Continuous variables were checked for normal distribution. The independent two-sample Student’s t-test was used for comparison of continuous variables between two different groups. Mean differences between several groups were compared using univariate ANOVA (Turkey-HSD post-hoc test). Dichotomous variables were compared using the χ^2^ test. Diagnostic performance of different measurement approaches was primarily analysed by plotting receiver operating characteristics and comparing the area under the curve (AUC). AUCs were compared using the method by DeLong et al. [20]. A *p* value of less than 0.05 was considered significant. A subgroup of 20 LLC-positive patients was investigated to assess inter- and intra-observer agreement regarding the different mapping measurement approaches according to the method of Bland and Altman.

## Results

### Clinical characteristics

The patient group was 38 ± 16.3 years and 77% were males. The healthy control group was 36.9 ± 13.5 years and 74% were males. LVEF was significantly lower in patients compared to healthy controls (55.3 ± 9.4% vs. 61.6 ± 4.6%; *p* = 0.004). No significant differences could be detected regarding heart rate, LVEDV/body surface area (BSA), and IVST between patients and healthy controls (*p* > 0.05 for all parameters). Troponin I, as well as creatine kinase were pathologically elevated in all patients. Further, blood levels of leucocytes and C-reactive protein were significantly higher in patients (10.4 ± 4.7 10^3^/μmL and 70.7 ± 99 mg/L) compared to healthy controls (6.5 ± 1.7 10^3^/μmL and 1.0 ± .8 mg/L, *p* ≤ 0.01 respectively) (Table 1).

**Table 1:** Patients’ clinical and CMR characteristics

	Healthy Control Group (*n* = 30)	Myocarditis Group (*n* = 50)	*p* Value
Age (y)	36.9 ± 13.5	38 ± 16.3	0.764
Male (%)	23 (77)	37 (74)	0.505
BMI (kg/m^2^)	25.3 ± 4.1	25.4 ± 5.2	0.937
CMR results			
Symptom onset to CMR (d)	–	2.9 ± 1.8	–
Heart rate (bpm)	68.1 ± 12.4	74.1 ± 15.2	0.170
LVEF (%)	61.6 ± 4.6	55.3 ± 9.4	0.004
LVEDV/BSA (ml/m^2^)	74.3 ± 9.4	78.1 ± 17.1	0.552
IVST (mm)	9.5 ± 1.6	9.8 ± 1.7	.311
Blood values			
Max. Trop I (ng/ml)	–	18.9 ± 78.2	–
Leucocytes (10^3^/μmL)	6.5 ± 1.7	10.4 ± 4.7	0.001
C-reactive protein (mg/L)	1.0 ± .8	70.7 ± 99	<0.001
CK-MB mass	–	21.4 ± 36.6	–
Creatine kinase (U/L)	–	363.5 ± 489.8	–
Haematocrit (%)	41.7 ± 3.9	40.5 ± 5.6	0.304
Number of patients with myocarditis specific parameters (%)			
Fulfilling the LLC (%)	0 (0)	36 (72)	<0.001
T2 ratio/visible edema (%)	2 (7)	33 (66)	<0.001
EGEr/aME (%)	6 (20)	24 (59)	0.001
LGE (%)	0 (0)	39 (78)	<0.001
Myocarditis specific parameters			
T2 SI myocardium	763.5 ± 268.9	893.4 ± 262.7	0.004
T2 SI skeletal muscle	472.5 ± 157.3	465 ± 132.8	0.826
T2 ratio	1.6 ± 0.3	1.9 ± 0.4	<0.001
EGEr	2.3 ± 1.9	3.1 ± 1.8	0.063
aMe (%)	37.5 ± 18.3	54.4 ± 31.6	0.007
LGE in %	0	12,1 ± 11.8	–

*BMI* body mass index, *CK* creatine kinase, *CMR* cardiovascular magnetic resonance, *EGEr* early gadolinium enhancement ratio, *LGE* late gadolinium enhancement, *LLC* Lake Louise Criteria

A group of patients showing no visible edema or LGE (CMR negative) was specifically examined (*n* = 9). Patients were 44 ± 18 years and 56% were male. Neither LVEF (58.3 ± 9.8%), nor LVEDV/BSA (81.2 ± 27.2 ml/m^2^) or IVST (10 ± 2 mm) showed significant differences compared to the healthy control group (p > 0.05). Leukocytes were within normal range (9.8 ± 2.6 10^3^/μmL) and the Troponin I level was only mildly elevated (0.4 ng/ml). Creatine kinase accounted for 260.4 ± 235.1 U/L and C-reactive protein for 12.8 ± 28 mg/L. Patients’ characteristics are listed in Table 1.

### CMR characteristics

CMR was performed on average 2.9 ± 2.2 days (1–8 days) after onset of symptoms. The majority (*n* = 39, 78%) of patients had LGE in a non-ischemic pattern. In these cases, LGE accounted for 12% of the LV mass (range 2–51%). A majority (*n* = 33, 66%) also showed an increased T2 ratio and/or visible edema. The segmental distribution of LGE and visible edema is shown in Additional file 1. A total of 24 patients (59%) presented with either significantly increased EGEr or absolute myocardial enhancement (aMe). There were 36 (72%) patients who fulfilled the classic LLC whereas 42 (84%) patients fulfilled the updated LLC. Patients’ CMR characteristics are listed in Table 1.

### Measurement results

All measurement approaches (global, mSAX, ConSept, and remote) revealed significantly higher T1 and T2 relaxation times as well as ECV values in the respective myocardial regions in patients compared to healthy controls (*p* < 0.05 for all parameters). The global measurement approach showed highest diagnostic performance regarding all mapping parameters (AUCs, native T1: 0.903, post-contrast T1: 0.608, T2: 0.847, ECV: 0.731). Direct comparison of the different measurement approaches revealed significant differences in diagnostic performance between the global and the remote approach regarding T1 relaxation times and ECV (*p* = 0.001 and *p* = 0.002 respectively). Further, the global measurement approach revealed significantly higher T1 relaxation times compared to the ConSept approach (AUCs: 0.903 vs. 0.783; *p* = 0.003) and nearly significant differences compared to the mSAX approach (AUC: 0.850; *p* = 0.051).

**Fig. 3:**
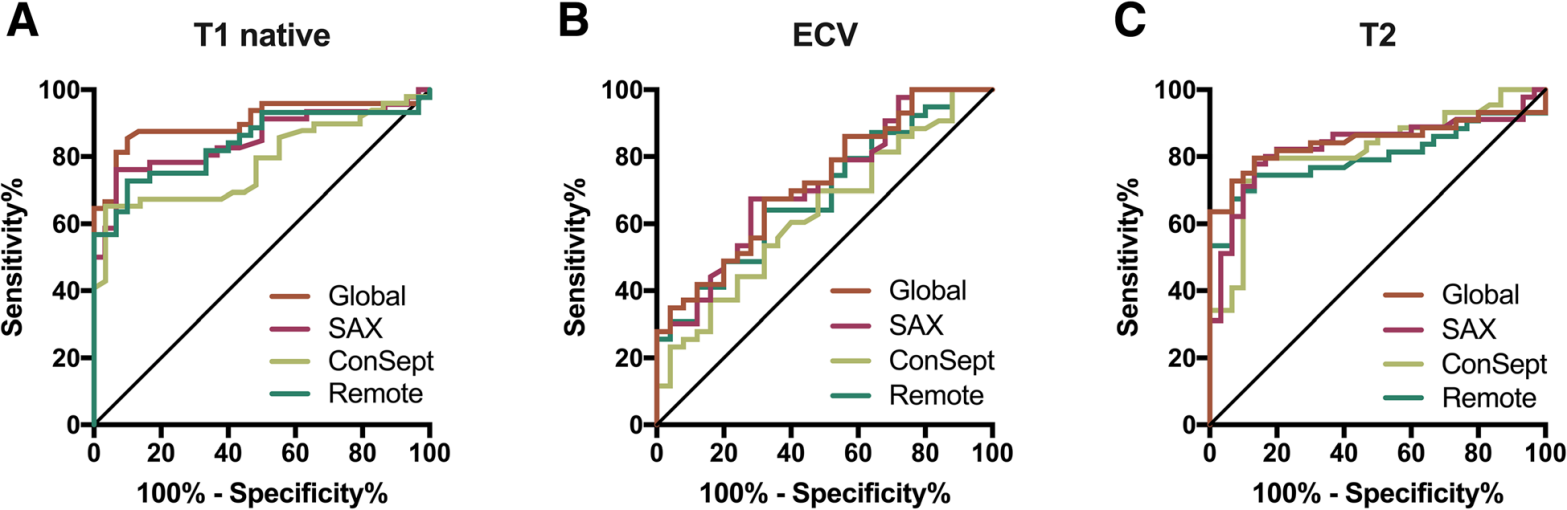
Receiver operator characteristic curves for the different measurement approaches. The graphs show receiver operator characteristic curves (ROCs) for the different measurement approaches. (**a**) Native T1: global (AUC: 0.903), mSAX (AUC: 0.850), midventricular septal (ConSept) (AUC: 0.783), remote (AUC: 0.841). (**b**) ECV: global (AUC: 0.731), mSAX (AUC: 0.717), ConSept (AUC:0 .633), remote (AUC: 0.685). (**c**) T2: global (AUC: 0.847), mSAX (AUC: 0.831), ConSept (AUC: 0.820), remote (AUC: 0.799)

T2 relaxation times showed no significant differences between all measurement approaches (*p* > 0.05 for all parameters). Measurement results and diagnostic performance of the different measurement approaches are listed in Tables 2 and 3 as well as Fig. 3. The distribution of T1 and T2 relaxation times within the LV is displayed in the Additional file 2.

**Table 2:** Results for quantitative CMR parameters regarding different measurement approaches

	Control group	Myocarditis group	pValue
Native T1 (ms)			
Global	958.9 ± 22.5	1027.2 ± 49.3	<0.001
mSAX	954.3 ± 28.6	1023.4 ± 29.9	<0.001
ConSept	969.7 ± 28.6	1022.8 ± 60.8	<0.001
Remote	958.9 ± 22.5	1014.3 ± 54.7	<0.001
ECV (%)			
Global	27.7 ± 3.2	32 ± 6.4	0.010
mSAX	26.6 ± 3.5	31.4 ± 7.2	0.005
ConSept	27.7 ± 4.2	31 ± 7.2	0.043
Remote	27.7 ± 3.2	31.9 ± 7.1	0.027
T2 (ms)			
Global	51.6 ± 1.9	58 ± 6	<0.001
mSAX	51.4 ± 3.2	58.4 ± 7	<0.001
ConSept	51.2 ± 3.9	58.2 ± 7.4	<0.001
Remote	51.6 ± 1.9	56.4 ± 6.1	<0.001

*ConSept* midventricular septal wall, *ECV* extracellular volume fraction, *mSAX* midventricular short axis

**Table 3:** Results for diagnostic performance of different measurement approaches

	Cut off	AUC	Sensitivity (%)	Specifity (%)	PPV (%)	NPV (%)	Accuracy (%)
Native T1 (ms)							
Global	> 980	0.903	85	90	93	79	87
mSAX	> 985	0.850	76	93	95	72	83
ConSept	> 999	0.783	65	97	97	62	77
Remote	> 980	0.841	73	90	91	69	80
ECV (%)							
Global	> 31	0.731	47	88	87	49	62
mSAX	> 28	0.717	67	72	81	56	69
ConSept	> 27	0.633	70	52	71	50	63
Remote	> 29	0.685	64	68	76	55	66
T2 (ms)							
Global	> 54	0.847	80	87	90	74	82
mSAX	> 53	0.831	78	87	90	72	81
ConSept	> 53	0.820	80	87	90	74	82
Remote	> 54	0.799	74	87	89	70	79

*ECV* extracellular volume fraction, *mSAX* midventricular short axis, *NPV* negative predictive value, *PPV* positive predictive value

The global measurement approach showed highest intra- and inter-observer agreement regarding the commonly used measurement approaches as shown in Table 4 (corresponding box-plots are shown in Additional file 3). The *p*-value regarding linear correlation was <0.002 for all comparisons.

**Table 4:** Results for intra- and inter-observer agreement regarding the different measurement approaches

	Intra-observer agreement			Inter-observer agreement		
	Δm (ms)	95% CI (ms)	Pearson’s r	Δm (ms)	95% CI (ms)	Pearson’s r
Native T1						
Global	4.9	[−8.2;18.1]	.99	3.2	[−14.6;20.9]	.98
mSAX	8.1	[−23;39.3]	.96	0.9	[−33.5;35.4]	.96
ConSept	11.8	[− 46.9;70.6]	.85	17	[−46.8;80.8]	.83
Remote	4.2	[−9.9;18.3]	.99	5.6	[− 34.8;46]	.88
ECV						
Global	0.6	[−3.8;5.1]	.96	0.5	[−3;4]	.98
mSAX	2.1	[−6.4;10.6]	.87	1.8	[−5.6;9.2]	.90
ConSept	2	[−5.7;9.8]	.89	1.4	[−5.6;8.4]	.92
Remote	0.5	[−2.9;3.8]	.98	0.6	[−1.9;3.2]	.99
T2						
Global	0.5	[−2;3]	.97	0.3	[−3;3.5]	.97
mSAX	0.7	[−7;8.3]	.88	−0.2	[−7.1;6.7]	.94
ConSept	2.2	[−3.9;8.2]	.92	3.1	[−7.4;13.6]	.73
Remote	0.7	[−1.4;2.7]	.99	0.5	[−2.9;3.8]	.97

## Discussion

To our knowledge this is the first study comparing all established mapping measurement approaches in the same cohort of patients with clinically suspected acute myocarditis. Our main findings were: 1) All measurement approaches (global, mSAX, ConSept) allowed for reliable distinction between healthy and diseased myocardium, irrespective of the mapping technique used; 2) even measurements in the remote myocardium reliably differentiated healthy from myocarditis patients; 3) the global measurement approach showed overall the highest diagnostic performance.

### Comparison of mapping measurement approaches

#### Native T1

Native T1 relaxation times reflect information from intra- and extracellular space, thus allowing for both, detection of myocardial edema and fibrosis. Native T1 has further shown to be an excellent discriminator between healthy and myocarditis patients, even exceeding the diagnostic performance of the standard LLC [8]. Unlike T2 relaxation times native T1 relaxation times show significant regional differences in healthy human myocardium with extremes between septal and lateral segments. Septal values are higher and show smaller spread of values [21]. This led to the concept of measuring native T1 relaxation times only within the midventricular septum (ConSept approach). Compared to the mSAX-approach, which was used for native T1-measurement in most published studies at that time, ConSept proved to be a robust technique which allowed for better discrimination between healthy and diseased myocardium in patients assumed to have diffuse myocardial disease [13]. Further, ConSept was successfully applied in patients with cardiac sarcoidosis using native T1 and T2 mapping [22]. In agreement with previous studies, healthy controls in the underlying study showed higher native T1 relaxation times within the midventricular septum compared to lateral segments. However, owing to the typical pattern of disease, predominantly affecting the midventricular lateral and infero-lateral segments, ConSept showed the weakest diagnostic performance of all measurement approaches in the current study. Ultimately, the fact, that the midventricular septum is usually least involved in the disease process resulted in the highest specifity and positive predictive value for ConSept compared to the other measurement approaches. Although, lateral segments are usually predominantly affected in acute myocarditis, inflammatory processes still involve the whole left ventricle. Thus, it is reasonable that the global approach, which incorporates the entire left ventricular myocardium, represented the most accurate method of T1 measurement. The performance of the mSAX and the remote approach can be explained accordingly: mSAX, including midventricular lateral segments, showed the second best diagnostic performance and the remote approach, covering a majority of the LV myocardium, revealed the third best performance.

#### ECV

ECV represents a reliable marker for myocardial remodelling and fibrosis respectively and has proven to reliably differentiate between myocarditis patients and healthy subjects in numerous previous studies [5, 8, 23–26]. Further, it could be shown that in combination with LGE, ECV improved the diagnostic accuracy in patients with subacute, severe myocarditis compared with the standard LLC [25].

In the underlying study, all measurement approaches showed only moderate diagnostic performance using ECV, again with best results obtained by using the global measurement approach. One of the reasons for these findings might be the short time interval between onset of symptoms and CMR in the current study. With an average time to CMR of 3 days, intracellular edema is most likely the predominant underlying pathophysiology [27] and the extent of interstitial edema might not suffice to allow for ECV elevation in diseased myocardium. It might also explain the variety of previous results for ECV regarding the diagnosis of acute myocarditis ranging from inability to differentiate health from disease to a diagnostic accuracy of 76% [25, 26]. Thus, the diagnostic yield of employing exclusively extracellular information for detection of myocarditis should be questioned.

#### T2

T2 relaxation times closely correlate with free tissue water content, thus making them an ideal marker for disease detection [8, 10–12, 23]. Further, T2 relaxation times have shown to be the only CMR parameter to allow for discrimination between acute and convalescent stages of myocarditis [23]. T2 relaxation times were significantly elevated in all segments compared with controls in the current study. As opposed to visible edema and LGE, which showed distinctive differences in regional distribution towards midventricular and basal infero-lateral segments, T2 relaxation times where overall evenly distributed in all patients’ segments with slight attenuation within apical inferior as well midventricular infero-lateral and inferior segments. Consequently, all measurement approaches showed a comparably high diagnostic performance with best result for the global approach. The fact that all patients’ segments showed markedly elevated T2 relaxation times shows the diffuse subclinical myocardial involvement of disease in the acute stage.

Our results regarding diagnostic accuracy of mSAX T1 (83%), T2 mapping (81%), and ECV (69%) are in line with previously published data by Lurz et al., who investigated the diagnostic performance of myocardial mapping using the mSAX approach versus endomyocardial biopsy in patients with suspected myocarditis (81%, 80%, and 75%, respectively) [28]. It should be noted, however, that these diagnostic accuracies are only valid in patients with acute symptoms. In patients with chronic symptoms the diagnostic performance of CMR is lower. According to the study of Lurz et al., only T2 relaxation times allowed for sufficient discrimination between patients with chronic symptoms and controls, which was explained by a shift in histological pathology during the course of the disease with diminishing free water content (T2:↓) and simultaneous expansion of extracellular space due to cellular debris and progression of diffuse fibrosis (native T1 and ECV:↔).

### Assessment of remote myocardium in acute myocarditis

The diagnostic value of T1 and T2 relaxation times as well as ECV in patients with clinically suspected acute myocarditis has been investigated extensively in the past [7, 8, 10–12, 23, 25, 26]. With few exceptions either using the mSAX approach or mid and basal SA slices for evaluation, most studies used the global measurement approach. Previous results uniformly showed that T1 and T2 mapping allow for reliable diagnosis of myocarditis and, furthermore differentiation between acute and convalescent stages of disease [7, 23]. However, a potential significant influencing factor that has not been elucidated until now, is that in previous studies T1 and T2 relaxation times were exclusively determined for areas with obvious myocardial damage (i.e. visible edema and/or LGE). This issue promoted us to additionally measure T1 and T2 relaxation times in patients’ remote myocardium where neither LGE, myocardial edema, nor regional wall motion abnormalities were visibly evident. Measurements within remote myocardium showed perfect agreement with the mSAX and the ConSept measurements, while the global approach, including also obviously affected segments, revealed significantly higher T1 relaxation times and ECV. Being able to differentiate healthy from diseased myocardium in patients with clinically suspected myocarditis only using remote myocardium allows for two conclusions: First, this finding once again proves whole LV involvement of disease despite a visual primarily focal/patchy appearance in CMR. Second, and in accordance with previous studies, it shows the superior diagnostic performance of myocardial mapping compared with STIR and LGE imaging.

### Mapping in patients with acute myocarditis but normal standard CMR

In our study, 9 patients with a clinical diagnosis of myocarditis were included, who neither showed LGE, nor visible edema and, thus were missed by the classic LLC. Although these patients did not show significant differences in LV function (i.e. LVEF, LVEDV/BSA, IVST) or semiquantitative imaging parameters (i.e. T2 ratio and aMe) compared to controls, native T1, T2, and ECV values were significantly higher using the global measurement approach. These results corroborate the findings by Ferreira et al. who were the first to show that native T1 is able to detect a significantly larger extent of myocardial injury compared to STIR imaging and LGE in this patient cohort [29]. In this study, it could be demonstrated that this is also the case for T2 relaxation times as well as ECV. Transferring our results to the LLC, inclusion of quantitative imaging parameters allowed for the CMR diagnosis of an additional 6 patients compared with the classic LLC. To our knowledge, this is the first study revealing an improved diagnostic performance of the updated LLC compared to the standard LLC.

### Which measurement approach to use in daily clinical routine?

Owing to an evenly distribution of T2 relaxation times within patients’ LV myocardium, all three measurement approaches revealed comparable diagnostic performances in detecting acute myocarditis using T2 mapping. This did not hold true for T1 mapping, where differences in diagnostic performances were present between the different measurement approaches, with best results for the global and the mSAX approach (Table 3). This raises the question, which method is best suited for disease detection in daily clinical routine.

Acquisition time and post-processing effort should not play a major role in decision making as the acquisition of one image slice accounts for one breath hold and also drawing of one vs. three endo- and epicardial contours accounts for less than two minutes when performed by an experienced examiner. It is rather the severity of disease as well as the time interval between onset of symptoms and CMR which decide about the added value of myocardial mapping in patients with myocarditis. As was shown in the subgroup of patients who did not fulfil the original LLK, both myocardial T1 and T2 mapping increased the diagnostic performance of CMR. In these patients, it is crucial to use the method with highest sensitivity and best reproducibility, which was the global approach. This is most likely also the case in subacute and convalescent stages of disease, however further studies are necessary to confirm this assumption. On the other hand, the mSAX and especially the ConSept approach could be used as an easier method to monitor convalescence of disease in case of follow-up examinations.

## Study limitations

As has been the case in almost all previously published CMR studies, diagnosis of myocarditis was based on validated clinical guidelines [14] rather than endomyocardial biopsy (EMB) as a reference standard. EMB is currently not performed in routine clinical practice due to its low sensitivity for ruling out myocarditis [30–33]. CMR diagnosis was performed based on both, the classic [34] and the updated LLC [4], which represent the international gold standard.

The results obtained in this study are only valid for patients with acute symptoms of myocarditis. In case of convalescent stages of disease, results may differ, as indicated above.

It has been shown that myocarditis coincides with atrial involvement [35]. In addition, it has recently been reported that the presence of atrial fibrosis in LGE imaging and T1 mapping is increased in patients with atrial fibrillation and seems to be associated with unfavourable outcome [36]. Also, T1 mapping has been reported to allow for assessment of myocardial remodelling [37, 38]. Both, atrial involvement and long-term evaluation of ventricular remodelling were not assessed in the underlying study, but should be investigated in further studies as they might provide incremental prognostic value.

## Conclusions

Native T1 and T2 mapping as well as ECV allow for accurate detection of disease in patients with acute myocarditis irrespective of the measurement approach used. Even measurements performed exclusively within remote myocardium allow for reliable detection of acute myocarditis, proving diffuse involvement of disease despite a mostly regional or patchy distribution pattern of visible pathologies. The global measurement approach provides the overall best diagnostic performance in acute myocarditis for both T1 and T2 mapping.

## Additional files

Additional file 1: Segmental distribution of LGE and visible edema. Segmental distribution of LGE (A) and visible edema (B) according to the 17 segment AHA-model. Values are presented in absolute numbers and percentage (listed in parentheses) of affected patients. (PNG 2869 kb)

Additional file 2: Segmental distribution of native T1 and T2 relaxation times as well as ECV. Segmental distribution of mean native T1 (A), ECV (B), and T2 relaxation times (C) of patients (red) and controls (black). (TIF 1562 kb)

Additional file 3: Bland-Altman plots showing intra (A)- and inter(B)-observer agreements of the different measurement approaches. (PPTX 444 kb)

## Data Availability

The datasets used and/or analysed during the current study are available from the corresponding author on reasonable request.

## Abbreviations

aMe: absolute myocardial enhancement
BSA: Body surface area
CI: Confidence interval
CMR: Cardiovascular magnetic resonance
ConSept: Conservative septal ROI measurement
ECG: Electrocardiogram
ECV: Extracellular volume fraction
EGEr: Early gadolinium enhancement ratio
EMB: Endomyocardial biopsy
GraSe: Gradient-spin-echo
HLA: Horizontal long axis
IVST: Interventricular septal thickness
LGE: Late gadolinium enhancement
LLC: Lake Louise criteria
LV: Left ventricle/left ventricular
LVEDV: Left ventricular enddiastolic volume
LVEF: Left ventricular ejection fraction
LVOT: Left ventricular outflow tract
mSAX: midventricular short axis
NPV: Negative predictive value
PPV: Positive predictive value
ROI: Region of interest
RV: Right ventricle/right ventricular
SAX: Short axis
STIR: Short tau inversion recovery
VLA: Vertical long axis
