# Supplementary figures and images for: Multiparametric cardiovascular magnetic resonance imaging in acute myocarditis: a comparison of different measurement approaches
Source: J Cardiovasc Magn Reson. 2019 Aug 29;21:54. doi: 10.1186/s12968-019-0568-x (PMC6714458; doi:10.1186/s12968-019-0568-x)

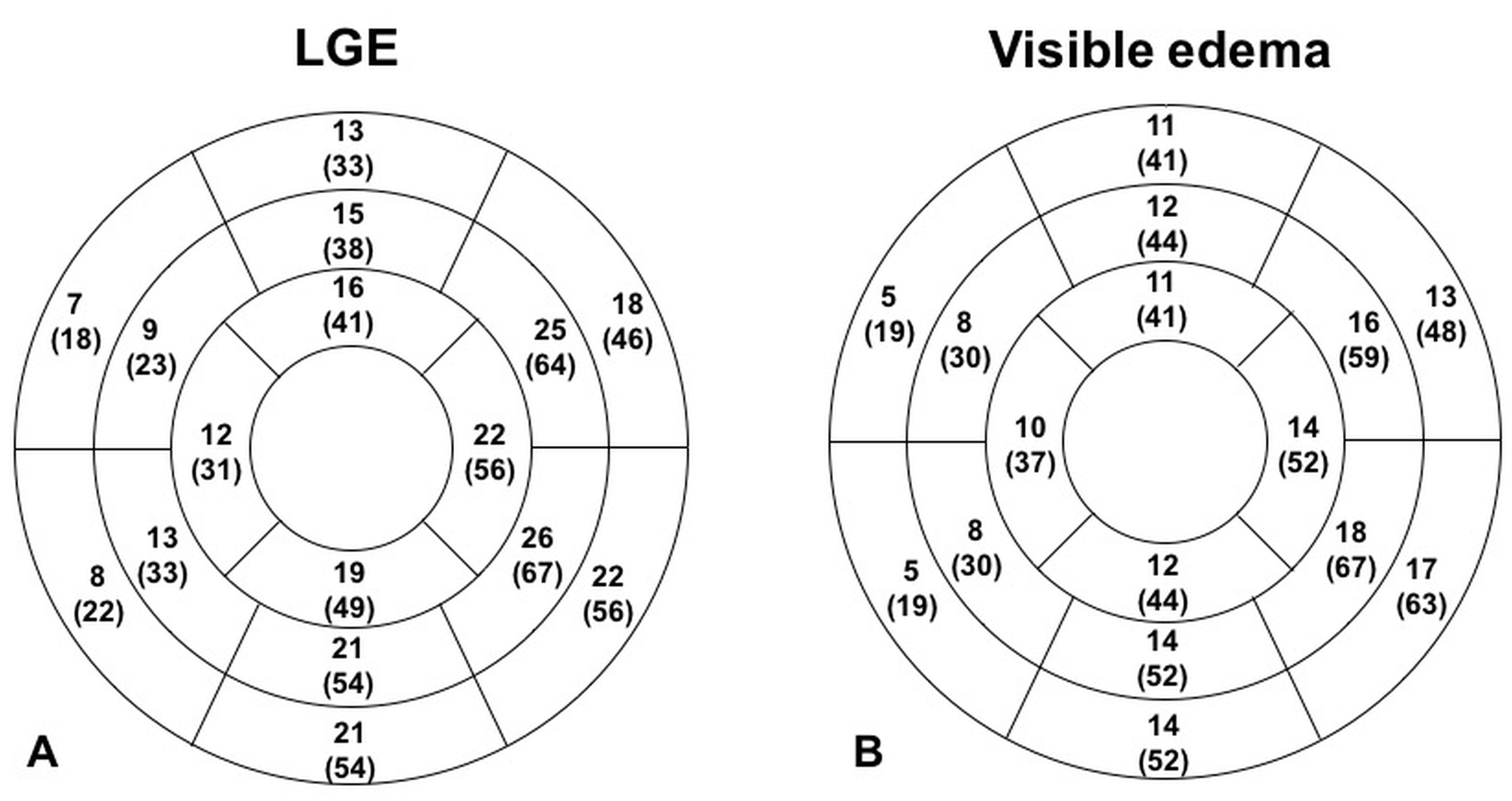

Supplement: Supplementary file 1 — Segmental distribution of LGE and visible edema. Segmental distribution of LGE (A) and visible edema (B) according to the 17 segment AHA-model. Values are presented in absolute numbers and percentage (listed in parentheses) of affected patients. (PNG 2869 kb) [file 12968_2019_568_MOESM1_ESM.png]

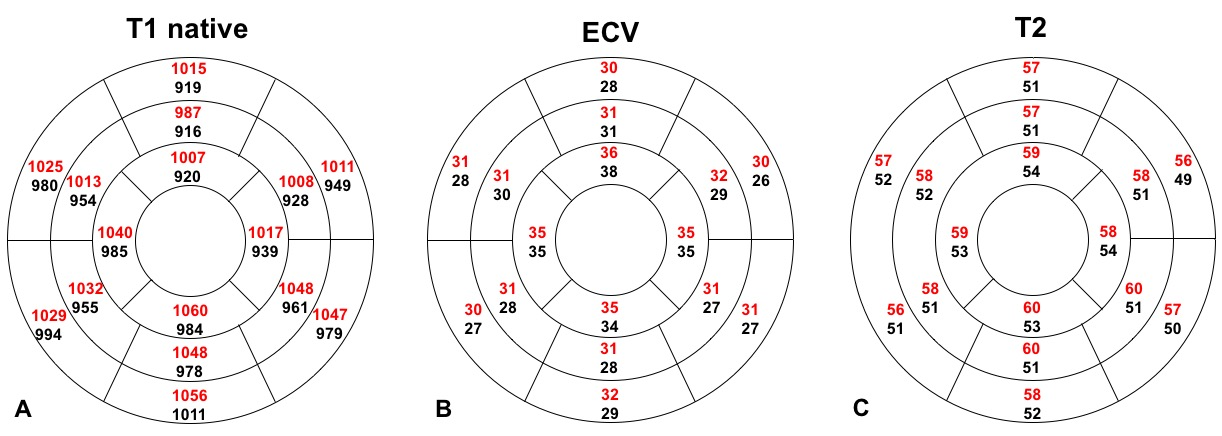

Supplement: Supplementary file 2 — Segmental distribution of native T1 and T2 relaxation times as well as ECV. Segmental distribution of mean native T1 (A), ECV (B), and T2 relaxation times (C) of patients (red) and controls (black). (TIF 1562 kb) [file 12968_2019_568_MOESM2_ESM.tif]

## Slide 1
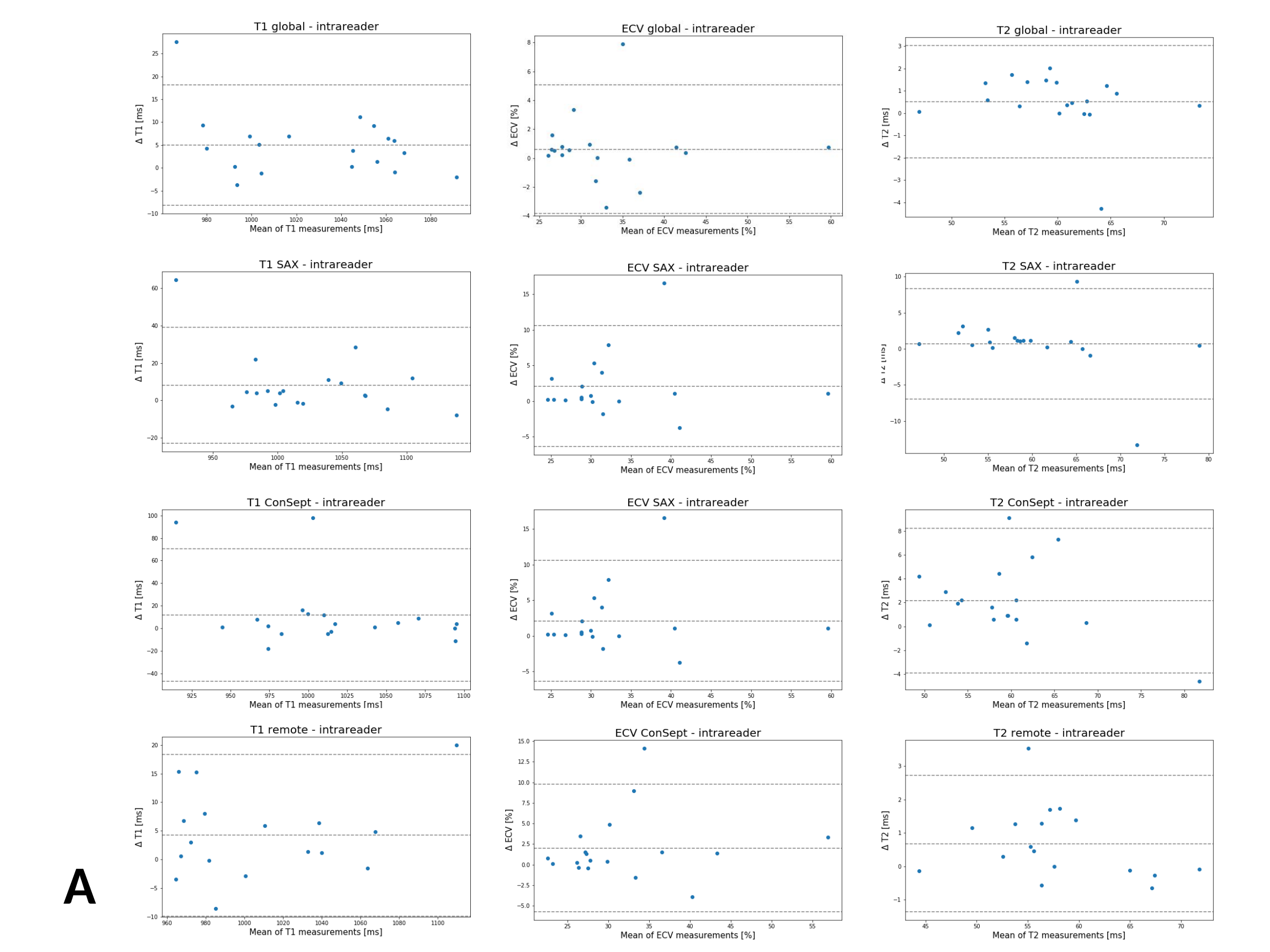

A

## Slide 2
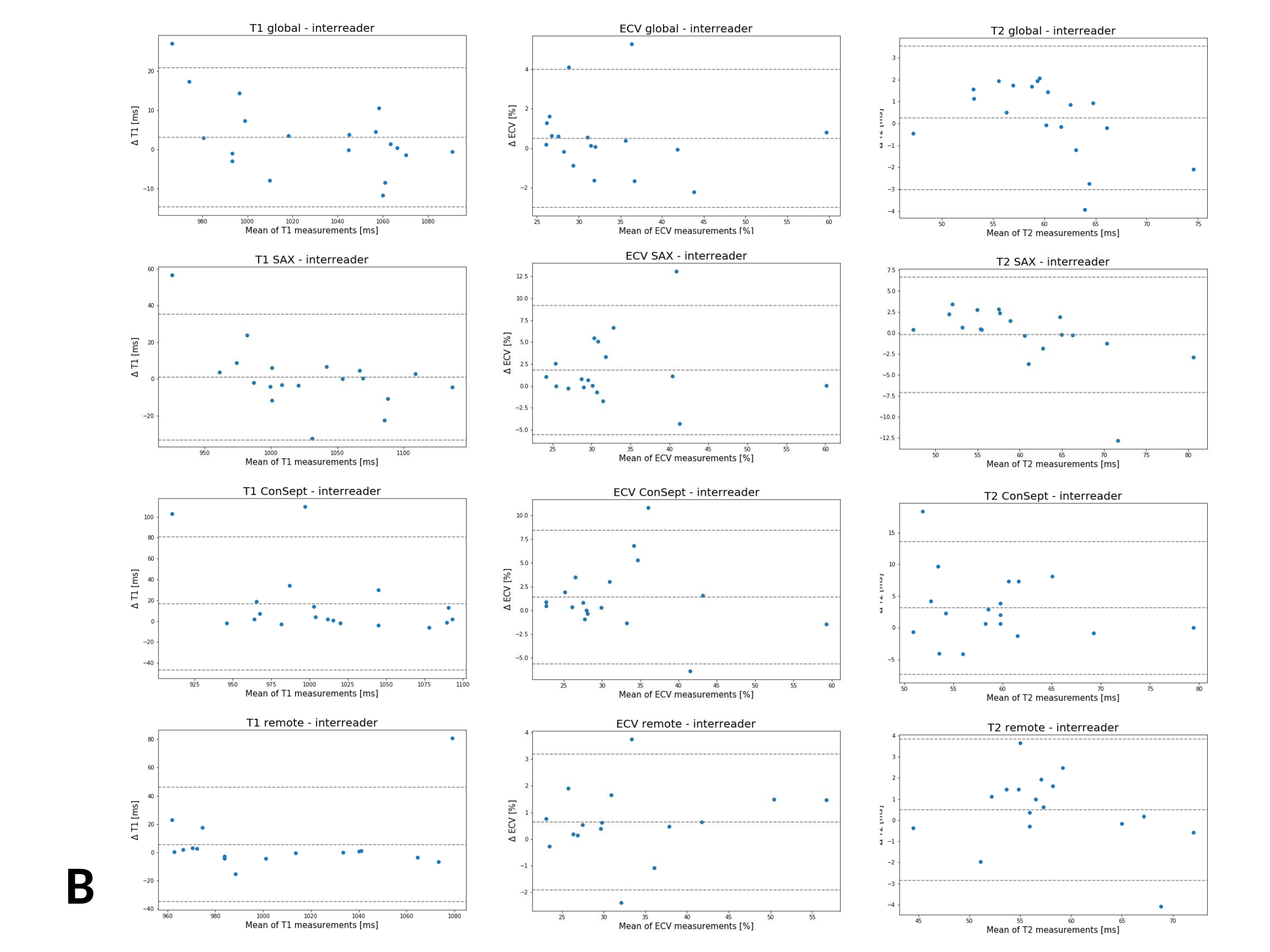

B

Supplement: Supplementary file 3 — Bland-Altman plots showing intra (A)- and inter(B)-observer agreements of the different measurement approaches. (PPTX 444 kb) [file 12968_2019_568_MOESM3_ESM.pptx]
